# Cyclic AMP Mimics the Anti-ageing Effects of Calorie Restriction by Up-Regulating Sirtuin

**DOI:** 10.1038/srep12012

**Published:** 2015-07-08

**Authors:** Zhuoran Wang, Lu Zhang, Yaru Liang, Chi Zhang, Zhiyu Xu, Lang Zhang, Ryosuke Fuji, Wei Mu, Liyuan Li, Junjun Jiang, Yong Ju, Zhao Wang

**Affiliations:** 1 MOE Key Laboratory of Protein Sciences, School of Medicine, Tsinghua University, Beijing 100084, P.R. China; 2 Department of Bioengineering, Graduate School of Bioscience and Biotechnology, Tokyo Institute of Technology, Tokyo 226-8051, Japan; 3 Department of chemistry, Tsinghua University, Beijing 100084, P.R. China

## Abstract

Cyclic adenosine monophosphate (cAMP) plays an important role in many biological processes as a second messenger, and cAMP treatment has been reported to extend the lifespan of wild-type *Drosophila melanogaster*. Our study showed that exogenous cAMP improved ageing-related phenotypes by increasing the protein level of Sirtuins, which prevented metabolic disorders to mimic the effect of calorie restriction. Experiments *in vitro* showed that cAMP directly bound to SIRT1 and SIRT3 and consequently increased their activity. These findings suggest that cAMP slows the ageing process and is a good candidate to mimic calorie restriction. Our research provides a promising therapeutic strategy to target metabolic disorder-induced ageing-related diseases.

## Introduction

Calorie restriction is an effective intervention to extend both the average and maximum lifespan^1^. However, the anti-ageing effect of calorie restriction calls for an approximately 30%–40% reduction in caloric intake, which is intolerable for most people. Many researchers have attempted to find a drug to mimic calorie restriction that has anti-ageing effects without reducing energy intake, such as resveratrol^2^. A recent study reported that resveratrol exerts anti-ageing effects by inhibiting cAMP hydrolysis. Rolipram, which specifically inhibits cAMP hydrolysis, had similar effects as resveratrol^3^, indicating the potential of cAMP as a candidate calorie restriction mimetic. Another study showed that the lifespan of wild-type *Drosophila melanogaster* significantly increased after cAMP treatment^4^, suggesting that cAMP may play a pivotal role in delaying the ageing process of organisms.

SIRT1 has been proposed as an anti-ageing protein, and its activation results in health benefits in multiple organisms^5^. It has been reported that cAMP responsive-element binding (CREB) deficiency reduces the expression of SIRT1; CREB directly regulates the transcription of *Sirt1* in neuronal cells by binding to *Sirt1* chromatin^6^. A recent study showed that cAMP activated calmodulin kinase kinase II (CaMKKII) to increase the AMPK phosphorylation level, thus promoting NAD^+^ production and SIRT1 activation^3^. Another study showed that the activation of the cAMP-PKA signalling pathway led to rapid SIRT1 phosphorylation, without changing the NAD^+^ level^7^. These findings highlighted the relationship between cAMP and SIRT1 in different pathways and suggested the potential of cAMP to be a specific activator of SIRT1 and mimic the anti-ageing effect of calorie restriction.

SIRT3, as a SIRT1 homologous deacetylase, is mainly located in mitochondria, where it reduces the amount of reactive oxygen species (ROS) responsible for inducing cell senescence^8^,^9^. In addition, there is an independent system to regulate the formation and degradation of cAMP in mitochondria to control ROS generation^10^,^11^, but whether cAMP can regulate SIRT3 to reduce oxidative stress has not been reported.

cAMP performs a variety of metabolic-related hormone signalling processes as a second messenger, and the cAMP response for many hormones becomes blunted with ageing^12^, suggesting that there should be an important role for cAMP in the regulation of the ageing process. Our study showed that administration of exogenous cAMP (dibutyryl cyclic adenosine, db-cAMP) increased the protein level of Sirtuin to mimic the anti-ageing effects of calorie restriction, including the prevention of metabolic disorders and improvement in ageing-related phenotypes. The results showed that cAMP could directly combine with SIRT1/SIRT3, suggesting that cAMP has an anti-ageing effect and is a good candidate for a calorie restriction mimetic.

## Results

### Exogenous cAMP Improves the Ageing-associated Phenotype in Aged Mice

**Figure 1:**
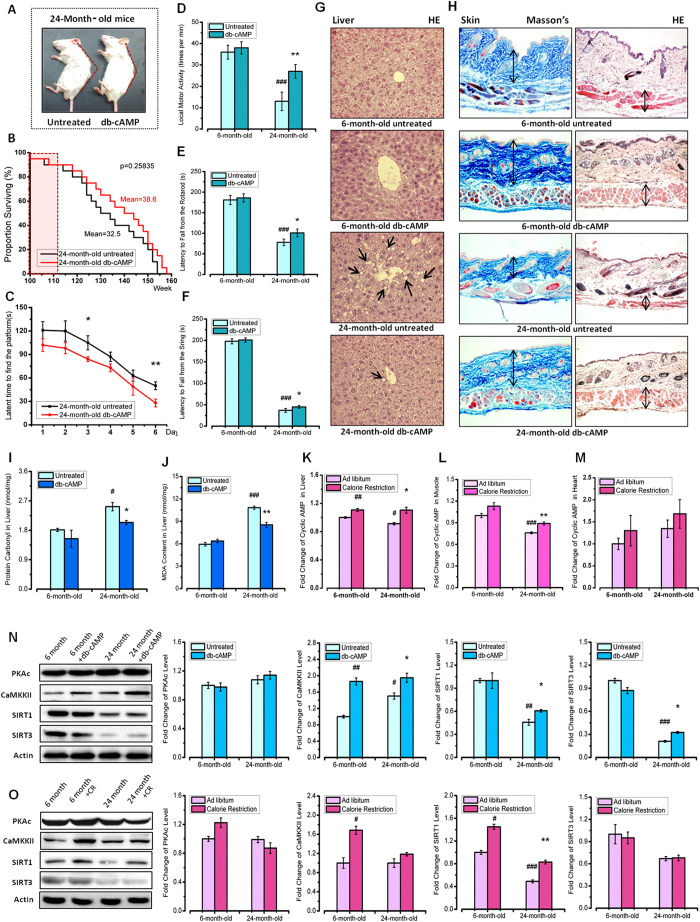
cAMP Treatment Mimics the Calorie Restriction Effect to Improve Ageing-associated Phenotypes in Aged Mice. (**A**) The appearance of aged mice (24-month-old) with (right) or without (left) db-cAMP treatment. The red line indicates the angle of the mouse spine. (**B**) The survival curve of aged mice with (red line) or without (black line) db-cAMP treatment. The red square indicated the administration period (12 weeks after 24-month-old) (n = 18–20 mice per group). (**C**) Morris water maze test result for mice with (red line) or without (black line) db-cAMP treatment. (**D**) Open field test result indicating the local motor activity. The data were captured by automatic detection. (**E**) Latency to fall from a rota-rod indicated the motor coordination of mice. (**F**) Latency to fall from a string indicated the muscle strength of mice. (**G**) HE staining for the liver of mice. The arrow indicates the hydropic degeneration phenotype. (**H**) HE and Masson’s staining for the skin of mice. The arrow indicates the thickness of collagen or muscle. Western blot results for the p-AMPK (Thr172) protein level in mice with or without calorie restriction treatment. (**I,J**) Protein carbonyl and MDA levels in liver tissue. (**K–M**) Fold change of cAMP level in mouse liver, muscle and heart with CR treatment. (**N**) Western blot results for PKAc, CaMKKII, SIRT1 and SIRT3 protein levels in the liver, with or without db-cAMP treatment (20 mg/kg db-cAMP). The gels were run under the same experimental conditions. (**O**) Western blot results for PKAc, CaMKKII, SIRT1 and SIRT3 protein levels in the liver, with or without calorie restriction treatment. The gels were run under the same experimental conditions. All the statistical analyses were performed using one-way ANOVA. ^#^p < 0.05, ^##^p < 0.01 ^###^p < 0.001 compared with untreated young mice; *p < 0.05, **p < 0.01, ***p < 0.001 compared with untreated aged mice.

To study the potential role of cAMP in the ageing process, we administered cAMP (20 mg per kg diet) to young (3-month-old) and aged (21-month-old) mice for 3 months. As observed from their appearance, cAMP treatment improved ageing-related phenotypes in aged mice, including thicker hair, stronger body and straighter spine (Fig. 1A). Compared with the untreated group, the average lifespan of cAMP treated mice was extended by 6 weeks (4%, Fig. 1B). Because cAMP signalling enhancers have been reported as a companion therapy in the treatment of cognitive dysfunction^13^, we performed a behavioural test, and the results showed, consistent with the mice’s appearance, that cAMP treatment improved behavioural performance in aged mice, including learning and memory, local motor activity, motor coordination, and muscular strength (Fig. 1C–F). As observed from tissue sections, liver hydropic degeneration was more severe in untreated aged mice compared with the cAMP-treated group (Fig. 1G). In the skin of aged mice, the collagen and muscle tissue was reduced, which could be restored by cAMP treatment (Fig. 1H). Because ROS accumulation is one of the causes of ageing-related phenotypes^14^, we assessed ROS-related damage to proteins and lipids in mouse livers and found that cAMP treatment can significantly reduce the amount of carbonyl and MDA levels in aged mice (Fig. 1I,J). These results explain why cAMP-treated mice exhibited improved appearance and show that cAMP treatment can delay the onset of ageing-related phenotypes.

### cAMP Mimics the Effects of Calorie Restriction by Raising Sirtuin Levels

It has been reported that starvation increases cAMP levels, which is the result of high levels of glucagon or low levels of insulin/IGF-1 signalling^15^. To study the relationship between cAMP and calorie restriction, we measured the cAMP level in different tissues and found that it was reduced in aged mouse liver and muscle tissue and showed significant increases after calorie restriction (Fig. 1K–M), suggesting that cAMP participates in calorie restriction. Then, we examined the levels of SIRT1 and SIRT3, both of which are key proteins associated with ageing and metabolism^16^. We found that cAMP treatment had a similar effect as caloric restriction on the elevation of SIRT1 and SIRT3 protein levels. Moreover, the protein level of SIRT6, which modulates telomeric chromatin^17^, was also decreased in aged mice but could be rescued by cAMP treatment (Figure S1A). The acetylation level of the pro-inflammatory transcription factor nuclear factor-kappa B (NF-κB) was increased significantly in aged mice; cAMP treatment reduced NF-κB, providing evidence that db-cAMP increases the specific activity of Sirtuin *in vivo,* as NF-κB has been reported to be deacetylased by Sirtuin^18^. CaMKKII levels increased after calorie restriction, whereas the PKAc level did not change significantly (Fig. 1N,O). These results indicated that in aged mice, cAMP has similar effects to calorie restriction on regulating Sirtuin, suggesting that cAMP mimics calorie restriction.

### cAMP Treatment Improves Adipose Metabolism to Mimic Calorie Restriction

**Figure 2:**
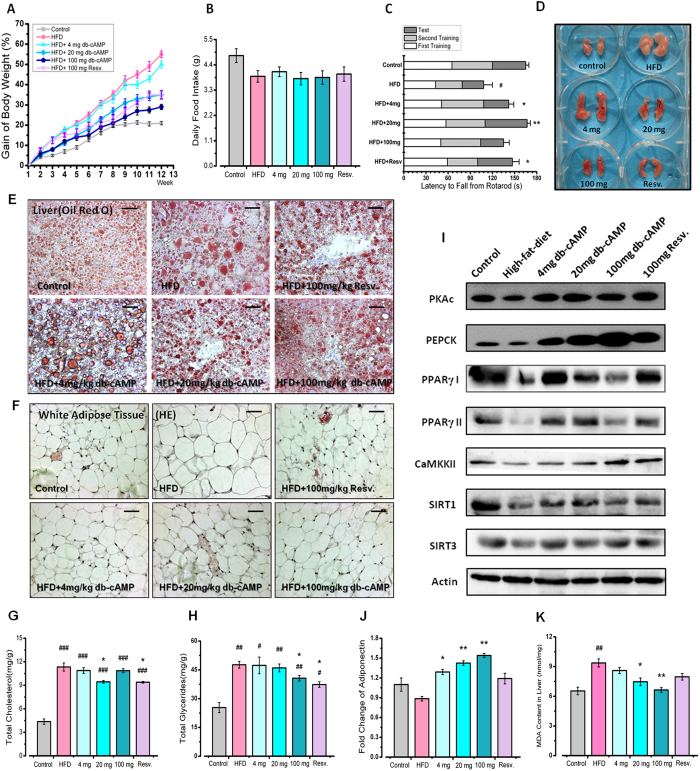
cAMP Treatment Improves Adipose Metabolism to Mimic Calorie Restriction. (**A**) The curve for the body weight gain. (**B**) Daily food intake. (**C**) Latency to fall from a rota-rod indicated the motor coordination of mice. (**D**) The photographs of epididymal adipose tissue. (**E**) Histological analysis of liver tissue, which was stained with oil red O to visualize lipid content and counterstained with haematoxylin. (**F**) Abdominal adipose tissue stained with haematoxylin and eosin. (**G**) The total cholesterol in mouse liver. (**H**) The total glycerides in mouse liver. (**I**) Western blot results for the PKAc, PEPCK, PPARγ, CaMKKII, SIRT1 and SIRT3 protein levels in treated or untreated mouse liver. The gels were run under the same experimental conditions. (**J**) Fold change in Adiponectin levels in liver. (**K**) MDA level in mice liver. All the statistical analyses were performed using one-way ANOVA. ^#^p < 0.05, ^##^p < 0.01 compared with control mice; *p < 0.05, **p <^ ^0.01, ***p < 0.001 compared with mice fed a high-fat diet.

To demonstrate the role of cAMP in calorie restriction, we fed mice a high-fat diet (60% energy from fat) containing different doses of cAMP or resveratrol as a positive control. The results showed that the body weights of cAMP-treated mice showed a dose-dependent reduction compared with the untreated mice fed the high-fat diet (Fig. 2A), with no significant changes in food intake (Fig. 2B). A high-fat diet led to a coordinated functional decline induced by over-weight; cAMP treatment improved motor coordination in high-fat-diet fed mice (Fig. 2C). We analysed the epididymal adipose tissue of mice and found that cAMP treatment caused a dose-dependent reduction in the size of adipose tissue (Fig. 2D). Biopsy results showed that a high-fat diet led to an increased size of adipocytes in mouse liver and abdominal adipose tissue; after cAMP treatment, the size of adipocytes trended towards normal (Fig. 2E,F). Triglycerides and total cholesterol levels were elevated in the high-fat-diet group compared with the untreated group, and the cAMP-treated group had lower levels of both, suggesting that cAMP could improve metabolic disorders (Fig. 2G,H).

Consistent with previous Western blot results in aged mice, SIRT1 and SIRT3 levels were improved to varying degrees after cAMP treatment. Over-expression of phosphoenolpyruvate carboxykinase (PEPCK) has been reported to extend the lifespan of mice^19^, and its level increased significantly in cAMP-treated mice. Peroxisome proliferator-activated receptor (PPARγ) promoted the differentiation of adipocytes and was also increased by cAMP treatment, which explained the decreased adipocyte size (Fig. 2I). It has been reported that adiponectin levels are reduced in obesity and diabetes^20^. Our data showed that the adiponectin level increased in a dose-dependent manner after cAMP treatment in high-fat-diet fed mice (Fig. 2J). These results suggested that cAMP treatment improves metabolic disorders by increasing SIRT1 and SIRT3 levels, suggesting that cAMP has the potential to be a calorie restriction mimetic.

### cAMP Promotes Sirtuin to Prevent Oxidative Damage and Cell Senescence

**Figure 3:**
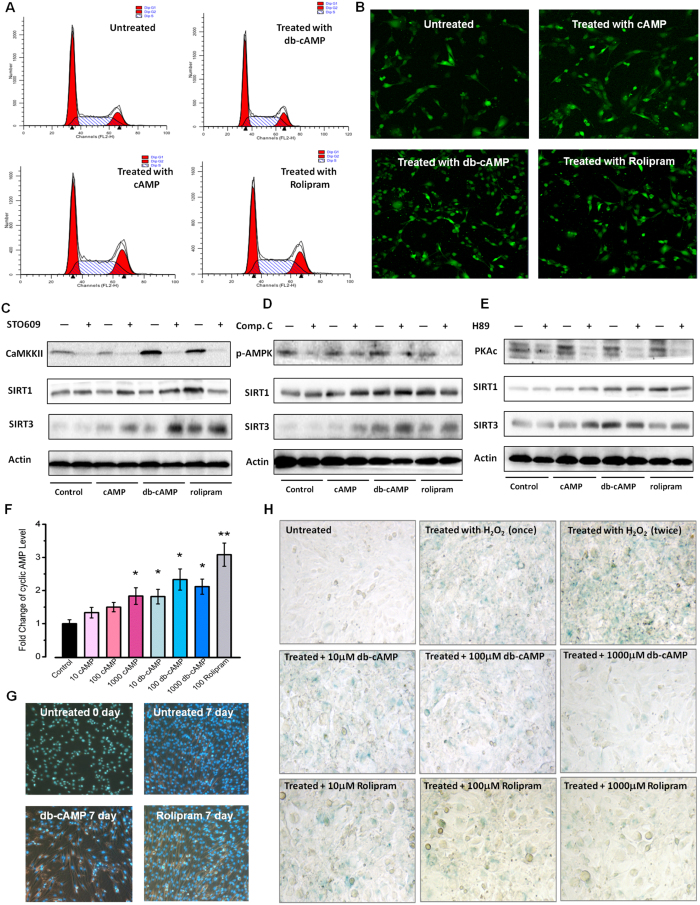
cAMP Promotes Sirtuin to Prevent Oxidative Damage and Cell Senescence. (**A**) Cell cycle detection by PI staining. (**B)** Fluo-3 staining for C2C12 cells. **(C**) Western blot results for the CaMKKII, SIRT1 and SIRT3 protein levels with or without the CaMKKII specific inhibitor STO609. cAMP, db-cAMP and Rolipram were also added (100 μM). (**D**) Western blot results for the AMPK, SIRT1 and SIRT3 protein levels with or without the AMPK-specific inhibitor Compound C. cAMP, db-cAMP and Rolipram were also added (100 μM). (**E**) Western blot results for the PKAc, SIRT1 and SIRT3 protein levels with or without the PKA-specific inhibitor H89. cAMP, db-cAMP and Rolipram were also added (100 μM). All of the gels (**C–E**) were run under the same experimental conditions. (**F**) Fold change of cAMP levels in cells treated with cAMP, db-cAMP (10, 100, 1000 μM) and Rolipram (100 μM). (**G**) Differentiation of C2C12 cells with or without db-cAMP or Rolipram treatment. Blue points indicate the nucleus of cells stained with Hoechst 33342. (**H**) SA-β-gal staining for C2C12 cells with or without H_2_O_2_ treatment. db-cAMP and Rolipram added at different concentrations (10, 100, 1000 μM). All the statistical analyses were performed using one-way ANOVA. *p < 0.05, **p < 0.01, ***p < 0.001 compared with untreated control group cells.

Decreased cell proliferation and increased oxidative stress are two important causes of cell senescence. To explore the mechanism underlying the anti-ageing effect of cAMP, we examined the changes in the cell cycle with or without cAMP treatment, compared with Rolipram as a positive control. The results showed that the proportion of S-phase cells exhibited an increasing trend but did not reach statistical significance, suggesting that the anti-ageing mechanism of cAMP did not depend on enhanced cell proliferation (Fig. 3A).

Studies have shown that Rolipram enhances cAMP levels and activates CaMKKII, which promotes AMPK phosphorylation and increases SIRT1 activity^3^,^21^. Therefore, we aimed to determine whether cAMP could also enhance the protein level of Sirtuin though the CaMKKII-AMPK pathway. We detected Ca^2+^ signalling and found that it was higher in cAMP- or Rolipram-treated cells (Fig. 3B). However, the results of our Western blot analysis showed that cAMP can also elevate SIRT1 and SIRT3 protein levels even during CaMKKII or AMPK inhibition (Fig. 3C,D). Our results showed that the Sirt1 mRNA level decreased in aged mice (Figure S3), and this decline was rescued by db-cAMP treatment. We concluded that the underlying mechanism was based on transcriptional regulation. Our results are also supported by previous studies that showed that cAMP responsive-element binding (CREB) deficiency reduces the expression of SIRT1^6^. Accordingly, we determined whether the effect of cAMP on SIRT1 was dependent on the PKA-CREB pathway. Interestingly, the Sirt1 mRNA level did not increase after db-cAMP or Rolipram treatment in cultured cells but did decrease after H89 (a specific PKA inhibitor) treatment. However, the protein levels of SIRT1 and SIRT3 were still enhanced by cAMP treatment even when PKAc was inhibited (Fig. 3E). Based on these findings, we believe that the regulatory mechanism of cAMP on SIRT1 levels is different in organisms and cultured cells. We hypothesized that cAMP could also regulate the protein level of SIRT1 post-transcriptionally via binding, which increases stability and prevents degradation of the SIRT1 protein.

To confirm the activating effect of cAMP on Sirtuin, we measured the intracellular cAMP levels and differentiation results in the C2C12 cell line (Fig. 3F,G) because glucose restriction inhibits skeletal myoblast differentiation by activating SIRT1^22^. The results showed that cAMP treatment inhibited the differentiation of C2C12 cells, suggesting that cAMP increased SIRT1 activity. Based on the results from our animal experiments, we speculated that the anti-ageing effect of cAMP may be achieved by reducing oxidative stress, so we used hydrogen peroxide (ROS generated) to induce cellular senescence. The results of senescence-associated β-galactosidase (SA-β-gal) showed that cAMP treatment can prevent ROS-induced cell senescence (Fig. 3H), indicating that cAMP reduces oxidative stress. The results also showed that db-cAMP treatment lost its function in reducing positive staining cell number under EX-527 (inhibitor of Sirtuin) treatment, indicating that the anti-oxidative stress ability of cAMP was dependent on Sirtuin (Figure S). These results suggested that the anti-oxidative effect of cAMP should depend on increasing the activity of both SIRT1 and SIRT3.

### cAMP Directly Binds with SIRT1 and SIRT3

**Figure 4:**
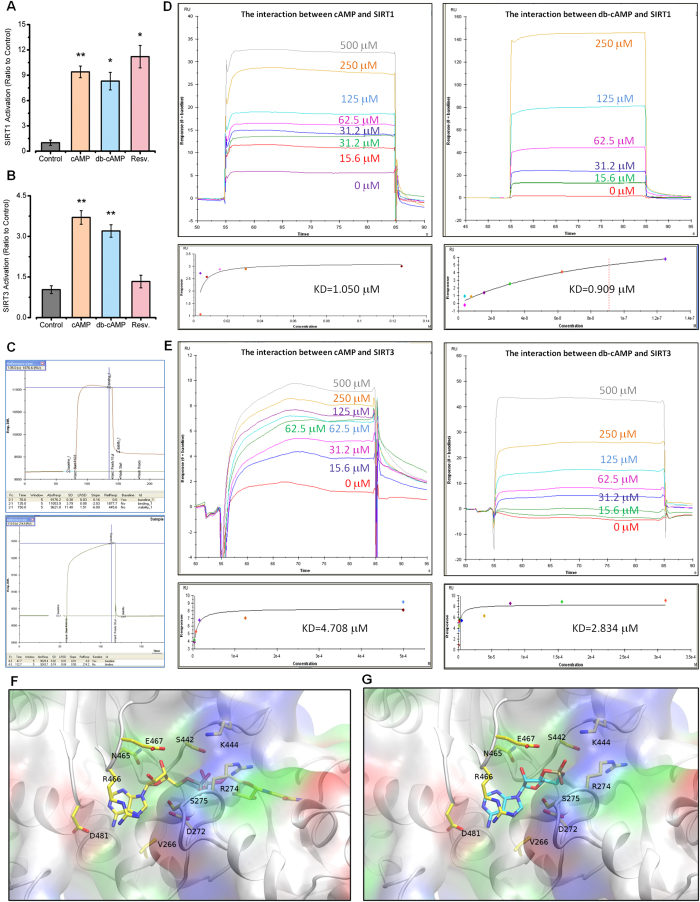
cAMP Directly Interacts with SIRT1 and SIRT3 *in vitro*. (**A**) SIRT1 activity analysis. (**B**) SIRT3 activity analysis. (**C–E**) Surface plasmon resonance measuring cAMP or db-cAMP binding to SIRT1 or SIRT3 proteins. SIRT1 or SIRT3 proteins were immobilized onto a CM5 sensor chip. Binding analyses were performed using a range of cAMP or db-cAMP concentrations (15.6–500 μM). (**F,G**) Mechanistic diagram of the interaction between cAMP and SIRT1 obtained by auto-dock analysis. The first graph shows the interaction between NAD and SIRT1 compared with the second graph, which shows the interaction between cAMP and SIRT1. All the statistical analyses were performed using one-way ANOVA. ^#^p < 0.05, ^##^p < 0.01 ^###^p < 0.001 compared with treated control group cells; *p < 0.05, **p < 0.01, ***p < 0.001 compared with untreated control group cells.

**Figure 5:**
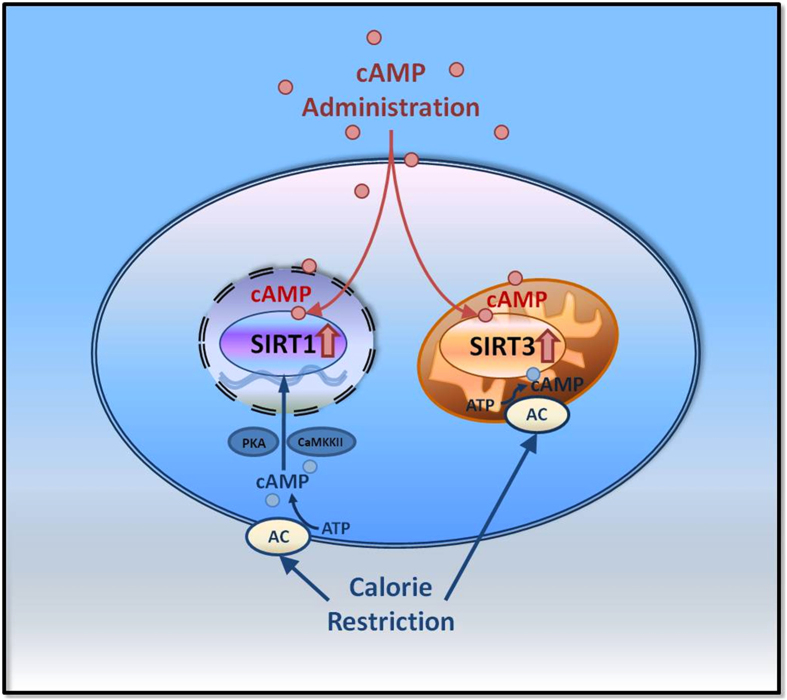
Graphical Abstract for the Underlying Mechanism of the cAMP Regulatory Effect on Sirtuin.

To further explore the mechanism of the cAMP regulatory effect on Sirtuin, we performed a Sirtuin activity assay *in vitro.* The results indicated that cAMP can increase SIRT1 and SIRT3 activities (Fig. 4A,B). Next, the surface plasmon resonance (SPR) technique was used to detect the interaction between cAMP and Sirtuin. The results showed that cAMP could directly bind with SIRT1 (KD = 1.05) and SIRT3 (KD = 4.708) *in vitro* (Fig. 4C–E). Because both SIRT1 and SIRT3 are NAD^+^-dependent lysine deacetylases and NAD^+^ contains an AMP moiety, the structural similarity of cAMP with NAD^+^ led to similar binding ability. Auto-dock analysis was performed to explain the mechanism underlying the cAMP binding result with SIRT1 and showed that the binding sites of cAMP were similar with NAD^+^ when they were bound on the SIRT1 protein (Fig. 4F,G). Our work could provide a starting point for a study on the underlying mechanism of the cAMP regulatory effect on Sirtuin (Fig. 5).

## Discussion

Our study shows that dietary cAMP supplements can up-regulate the protein level of Sirtuin to delay the onset of ageing-related phenotypes by preventing metabolic disorders. We demonstrated that cAMP could directly bind both SIRT1 and SIRT3 *in vitro*, increasing the activities of SIRT1 and SIRT3 and mimicking the effects of calorie restriction. These findings indicated that cAMP plays an important role in the ageing process and has promising potential as a calorie restriction mimetic to prevent ageing-related diseases.

Since the discovery of cAMP as a second messenger, which led to the researcher to win the Nobel Prize in 1971, studies focused on cAMP have been ongoing. However, until now, cAMP has not emerged as a candidate calorie restriction mimetic to delay the ageing process. Studies have shown that starvation will increase cAMP levels, which are the result of high levels of glucagon or/and low levels of insulin/IGF-1 signalling^15^,^23^ , suggesting that cAMP may play an important role in calorie restriction. However, exogenous cAMP has not been reported as a candidate calorie restriction mimetic before our study. Our work provides evidence supporting that calorie restriction increases the endogenous cAMP level in multiple organs to improve ageing-related phenotypes. Furthermore, exogenous cAMP can mimic the anti-ageing effect of calorie restriction, including the up-regulation of SIRT1 and SIRT3, both of which are key proteins in metabolism and ageing.

The physiological intracellular cAMP concentration is 0.1–1 μM, but in the case of hormones or stress, the concentration of cAMP can be increased by more than a hundred times (approximately 100 μM). In our assessment of SIRT1 and SIRT3 activities, we used 100 μM cAMP and db-cAMP to mimic concentrations within the physiological range. Because only a part of the supplemented cAMP could stably enter cells, we propose that these concentrations can have a similar effect as mild stress conditions, i.e., calorie restriction.

It was recently reported that resveratrol is a phosphodiesterase (PDE4) inhibitor, which competitively binds to PDE4 and inhibits cAMP degradation^3^. We speculated that cAMP may have the ability to bind to SIRT1, and the results of SPR proved our hypothesis and showed that cAMP and SIRT1 have a direct interaction *in vitro*. More interestingly, cAMP also has a direct interaction with SIRT3, which regulates mitochondrial ROS production and delays the ageing process. Both SIRT1 and SIRT3 are NAD^+^-dependent lysine deacetylases. NAD^+^ contains an AMP moiety, so cAMP directly interacts with the Sirtuin protein family. Our work provided evidence showing that cAMP could be a substrate analogue of Sirtuin under calorie-restricted conditions.

The auto-dock results showed that cAMP interacted with the NAD^+^ binding pocket of SIRT1 but did not block the catalytic site. The reaction of Sirtuin and NAD^+^ resulted in the hydrolysis of NAD^+^ into two parts, NAM and 2’-O-acetyl-ADP-ribose. Based on the analysis of the molecular structure of cAMP and NAD^+^, we could observe that when cAMP bound with SIRT1, only half of the binding pocket was occupied, and the catalytic side was available for the reaction between SIRT1 and NAD^+^. In this case, NAD^+^ will interact with the catalytic site, resulting in a higher risk of NAD^+^ hydrolysis. We hypothesized that the binding of cAMP on Sirtuin decreased the stability of NAD^+^, which improved the efficiency of the reaction between NAD^+^ and Sirtuin.

We hypothesized that the binding of cAMP and SIRT1 protected the catalytic site, which was the binding pocket of Sirtuin and NAD^+^. In this way, cAMP could increase the SIRT1 protein level post-transcriptionally as we have discussed previously. We speculate that the effect of cAMP on increasing the SIRT1 and SIRT3 protein levels was based on increasing their protein stability via binding, which could limit protein degradation.

Our work showed that cAMP is a good candidate to mimic calorie restriction. As an intracellular second messenger, cAMP is important in many biological processes. The ageing process is also complex and cannot be explained by just a single signalling pathway. We believe that the anti-ageing effect of cAMP may depend on a signal transduction network that involves more than a single target, meaning that cAMP targets multiple signalling cascades in the regulation of the ageing process.

### Experimental Procedures

#### Animals and Diets

Young (3-month-old) male BALB/c mice were purchased from Vital River Laboratories and treated according to the guidelines and with the approval of the Institutional Ethical Committee of China. Additional details are provided in the supplemental material.

#### Cell Culture and Treatments

C2C12 and 3T3-L1 cell lines were cultured in DMEM (Thermo Scientific) supplemented with 10% FBS in 5% CO_2_. Ex-527, Compound C, STO609 (Sigma) and H89 (Cell Signaling Technology) were added 1 hour prior to administration of cyclic AMP, db-cAMP and Rolipram (Enzo Life Sciences). Cells were then incubated for an additional 1 hour before being harvested. Additional details are provided in the supplemental material.

#### Behavioural Tests

Local motor activity and the time to fall from an accelerating rota-rod (Chinese Academy of Medical Sciences) were measured for each group to test motor coordination and balance. Morris water maze and latency to fall from a string tests were performed using the facilities in the Center for Life Science of Tsinghua University.

#### Carbonyl Content Measurement^8^

Protein carbonyls were quantified by 2,4-dinitrophenylhydrazine (DNPH). Briefly, 1 ml of 0.5 mg protein was treated with 200 ml of 10 mM of DNPH (dissolved in 2M HCl) for 1 hour, and then precipitated by 10% trichloroacetic acid. The pellets were washed with 1:1 (v/v) ethanol : ethyl acetate for three times, and solublized in 0.5 ml 0.2% SDS, 20 mM Tris-Cl, pH 6.8. Protein concentration in the final solution was then determined with a BCA kit (Thermo Scientific), and the absorbance at 360 nm was measured to calculate the carbonyl content. Protein samples treated with HCl but not with DNPH were used as blanks.

#### Nucleotide Measurements

The cAMP measurement was performed according to manufacturer’s instructions using the Glo-cAMP Assay Kit (Promega). Additional details are provided in the supplemental material.

#### Biochemical Measurements

Total cholesterol, triglyceride and MDA measurements were performed according to the manufacturer’s instructions using the quantification Kit (Blue Gene). SA-β-gal staining was performed according to the manufacturer’s instructions (Sigma).

#### Histochemical Section Observation

Liver and adipose tissue sections were stained by haematoxylin and eosin; skin collagen was stained by Masson’s Trichrome. Observation was performed using a light microscope (Leica) and captured at 40X magnification.

#### Protein Analyses

Samples for Western blotting were resolved on 10% SDS-polyacrylamide gels and transferred to PVDF membranes. The following antibodies were used: SIRT1, SIRT3 NF-κB, and PPARγ (Cell Signaling Technology) and CaMKKII, PKAc, SIRT6 (Abgent) and PEPCK, and Actin (Santa Cruz).

#### Surface Plasmon Resonance

Purified SIRT1 (Abcam, purity = 95%) or SIRT3 (R&D, purity = 95%) proteins were immobilized onto a CM5 sensor chip (T200 Biacore, GE). The pH sorting and immobilization results are shown in the supplementary information (Figure S3-4).

### Statistical Analyses

Comparisons between groups were made using one-way ANOVA. Differences were considered statistically significant at p < 0.05. Data analyses were performed using the statistical program Origin. In all experiments, error bars indicate standard error of the mean (SEM).

## Additional Information

**How to cite this article**: Wang, Z. *et al.* Cyclic AMP Mimic the Anti-ageing Effects of Calorie Restriction by Up-Regulating Sirtuin. *Sci. Rep.* **5**, 12012; doi: 10.1038/srep12012 (2015).

## Supplementary Material

Supplementary Information
