# Supplementary material for: Cyclic AMP Mimics the Anti-ageing Effects of Calorie Restriction by Up-Regulating Sirtuin
Source: Sci Rep. 2015 Jul 8;5:12012. doi: 10.1038/srep12012 (PMC4648391; doi:10.1038/srep12012)
Supplement: Supplementary Information [file srep12012-s1.pdf]

Supplemental Information

Cyclic AMP Mimics the Anti-ageing Effects of Calorie Restriction by Up-Regulating Sirtuin

Zhuoran WANG,[1, 4] Lu ZHANG,[1, 4] Yaru LIANG,[1] Chi ZHANG,[1] Zhiyu XU,[1] Lang ZHANG,[1] Ryosuke FUJI, [1, 2] Wei MU,[1] Liyuan LI,[1] Junjun JIANG,[1] Yong JU,[3] and Zhao WANG[1]*

[1] *MOE Key Laboratory of Protein Sciences, School of Medicine, Tsinghua University, Beijing 100084, P.R. China*

[2] *Department of Bioengineering, Graduate School of Bioscience and Biotechnology, Tokyo Institute of Technology, Tokyo 226-8051, Japan*

[3] *Department of chemistry, Tsinghua University, Beijing 100084, P.R. China*

[4]*These authors contributed equally to this work*

*Correspondence to: Dr. Zhao WANG

E-mail: zwang@tsinghua.edu.cn; Tel: 86-10-62772241

Supplemental Methods

Animals and Diets

All experiments were approved by the Institutional Ethical Committee of China (Animal Care and Use Committee). BALB/c mice were originally purchased from Vital River Laboratories (Charles River). Eight-week-old male mice were housed under a 12 hours light-dark cycle (light on 8 am–8 pm) with free access to food and water for the first month, until they reached 12 weeks of age. For all cAMP-related studies, mice were orally dosed daily with 4, 20, or 100 mg/kg (food) db-cAMP or with 100 mg/kg (food) resveratrol as a positive control. High-fat-diet fed mice were fed a diet containing 40% calories from fat (AIN-93, Research Diets) for up to 12 weeks. To measure the effect of cAMP as a calorie restriction mimetic, BALB/c mice were limited in food intake for 3 months with free access to water (60% weight of the daily food intake for each mouse). For studies involving aged mice with cAMP treatment, BALB/c mice had free access to food and water from 12 weeks until death, n=20 per group. However, some aged mice were sacrificed for different experiments, approximately n=3-5 per group.

Cell Culture and Treatments

C2C12 cells were seeded in cell culture plates (Corning) at 2.0–3.0 x10^4 cells/well in high-glucose DMEM growth medium supplemented with 10% foetal bovine serum and 1% pen/strep. After 24 hours, cells were switched to low serum media (2% horse serum, 1% pen/strep) to induce differentiation. The cells culture medium was changed

every 24 hours for 4 days. On the day of testing, 10, 100, or 1000 mM cAMP or db-cAMP, 100 mM of Rolipram or DMSO as a vehicle control was suspended in fresh DMEM media, and the cells were returned to the incubator for 6 hr with or without H89, EX527, STO609 or Compound C (Sigma). After 6 hr, cells were washed twice with 5 ml assay medium (un-buffered low-glucose DMEM supplemented with pyruvate and glutamine, pH 7.4).

Sirtuin Activity Measurements

The activity of Sirtuin was assessed according to the manufacturer's instructions using the quantification kit (Enzo Life Sciences). Initial deacetylation rates of SIRT1 or SIRT3 were determined at 25 μM Fluor de Lys SIRT1 and 25 μM NAD+ (37°C) in the absence (Control) or presence of 100 μM of the indicated compound. Reactions were stopped with Fluor de Lys® Developer II/2 mM nicotinamide, and fluorescence was measured (CytoFluor II, PerSeptive Biosystems, Ex. 360 nm, Em. 460 nm, gain=85).

Molecular Docking

The docking software was Autodock Vina, and the receptor protein was SirT-1 (PDB ID: 4I5I) using the crystal structure of chain A after removing the small molecule 4I5 and NAD. To add polar molecular hydrogen, the side chains of the receptor protein remained rigid during the docking process. Flexible ligand was defined plus polar hydrogen atoms to dock cAMP. The original body of the NAD docking area is the

centre of mass and molecules centred in a 26 × 26 × 26 angstrom cube area and included the entire molecule. The following Table S1 shows a different docking conformation binding energy, with the seventh conformation being used. The results showed that the binding mode of the NAD molecule is quite similar to the original.

Supplemental Figures and Legends

Figure S1. Db-cAMP Treatment Mimics the Calorie Restriction Effect to Improve Ageing-associated Phenotypes in Aged Mice. (A-C) The statistical analyses results of Figure 1G and 1H. **(D)** The mRNA level of Sirt1 in young and aged mice liver.

Figure S2. Db-cAMP Treatment Up-regulated Sirtuin in Aged Mice. (A) The prtein level of SIRT6. **(B)** The acetylation level of NF-κB.

Figure S3. cAMP Treatment Improves Adipose Metabolism to Mimic Calorie Restriction. (A) The statistical analyses of body weight. **(B)** Fold change of epididymal adipose tissue weight. **(C-F)** The statistical analyses results of Figure 2E, 2F and 2I.

Figure S4 cAMP Promotes Sirtuin to Prevent Oxidative Damage and Cell Senescence. (A) The statistical analyses of Cell cycle detection by PI staining. **(B)**The statistical analyses of Fluo-3 staining for C2C12 cells. **(C)** The mRNA level of Sirt1 for C2C12 cells. **(D)** SA-β-gal staining for C2C12 cells with or without H_2O_2 treatment.

Figure S5. The Immobilization Result of SIRT1 on CM5 chip for SPR analysis. (A) The pH sorting result for immobilization of SIRT1 on CM5 chip. **(B)** The immobilization result of SIRT1 on CM5 chip.

Figure S6. The Immobilization Result of SIRT3 on CM5 chip for SPR analysis. (A) The pH sorting result for immobilization of SIRT3 on CM5 chip. **(B)** The immobilization result of SIRT3 on CM5 chip.

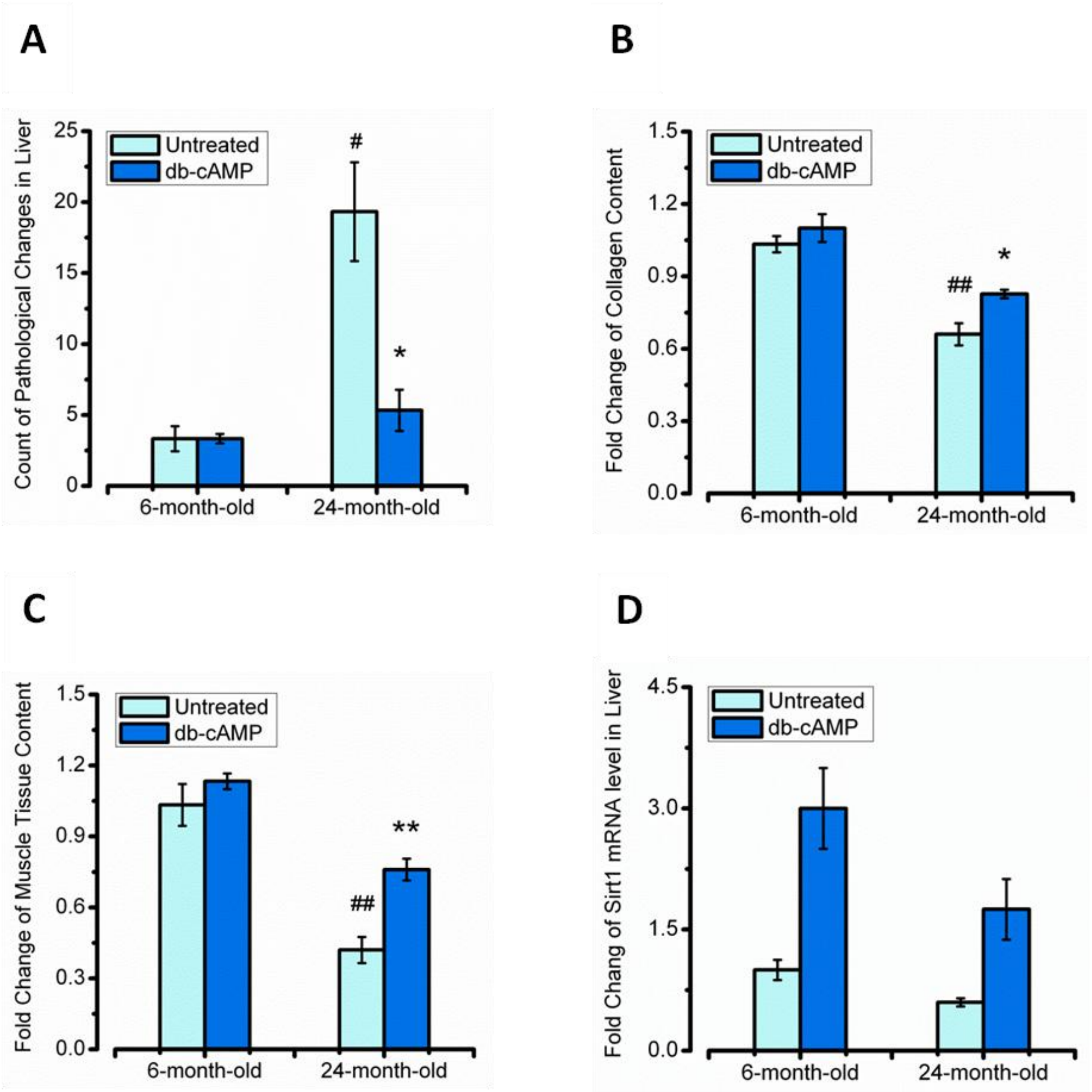

Figure S1. Db-cAMP Treatment Mimics the Calorie Restriction Effect to Improve Ageing-associated Phenotypes in Aged Mice.

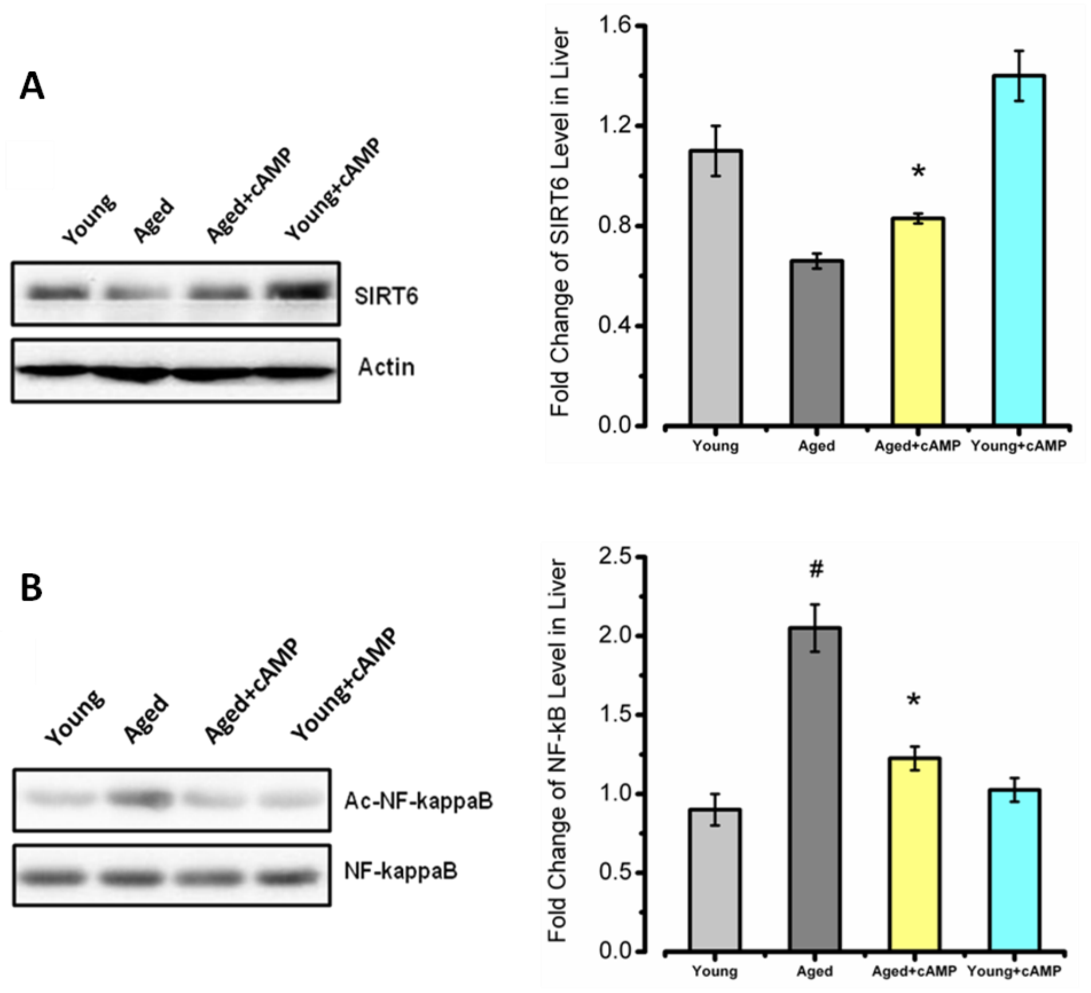

Figure S2. Db-cAMP Treatment Up-regulated Sirtuin in Aged Mice.

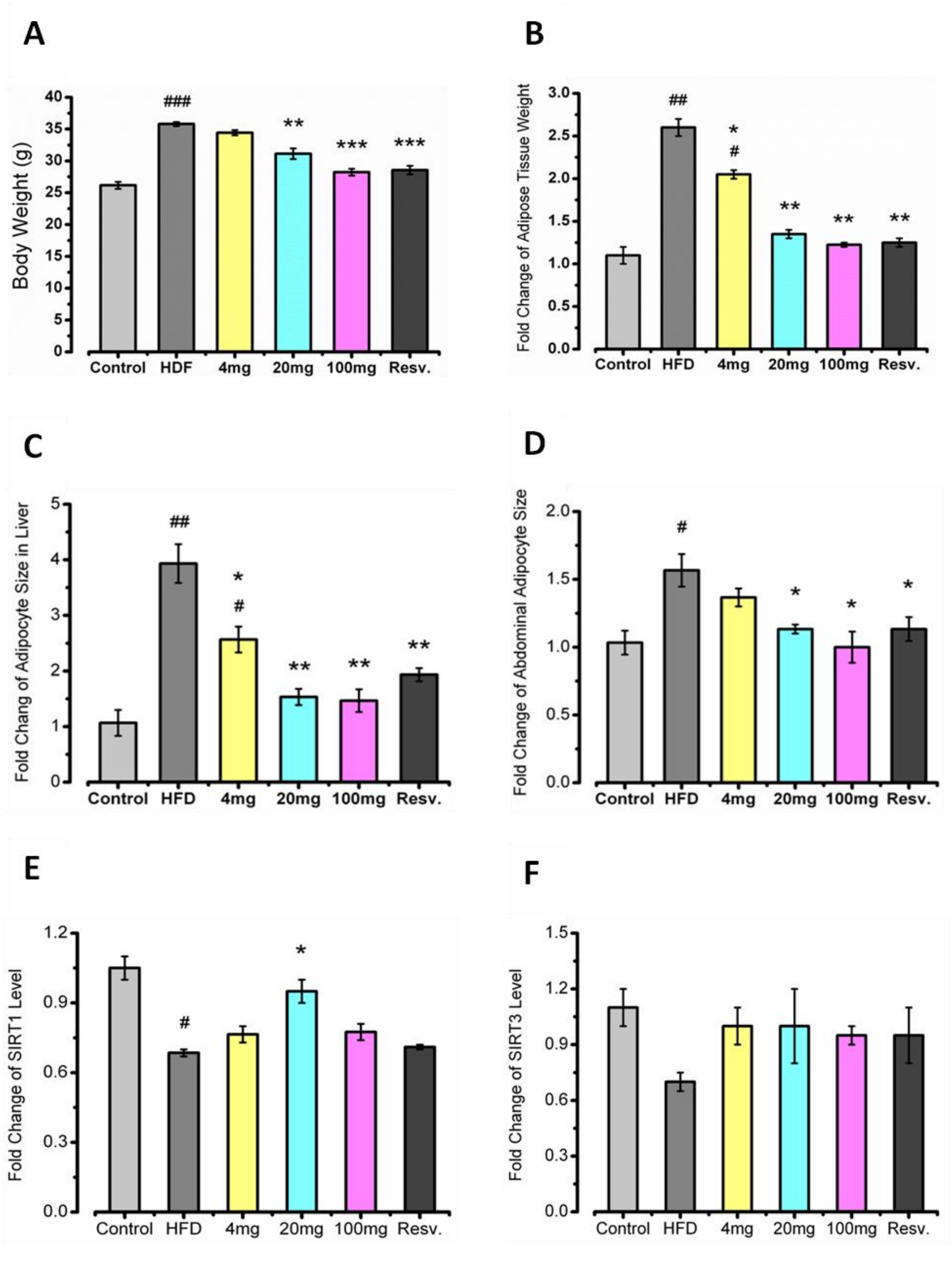

Figure S3. cAMP Treatment Improves Adipose Metabolism to Mimic Calorie Restriction.

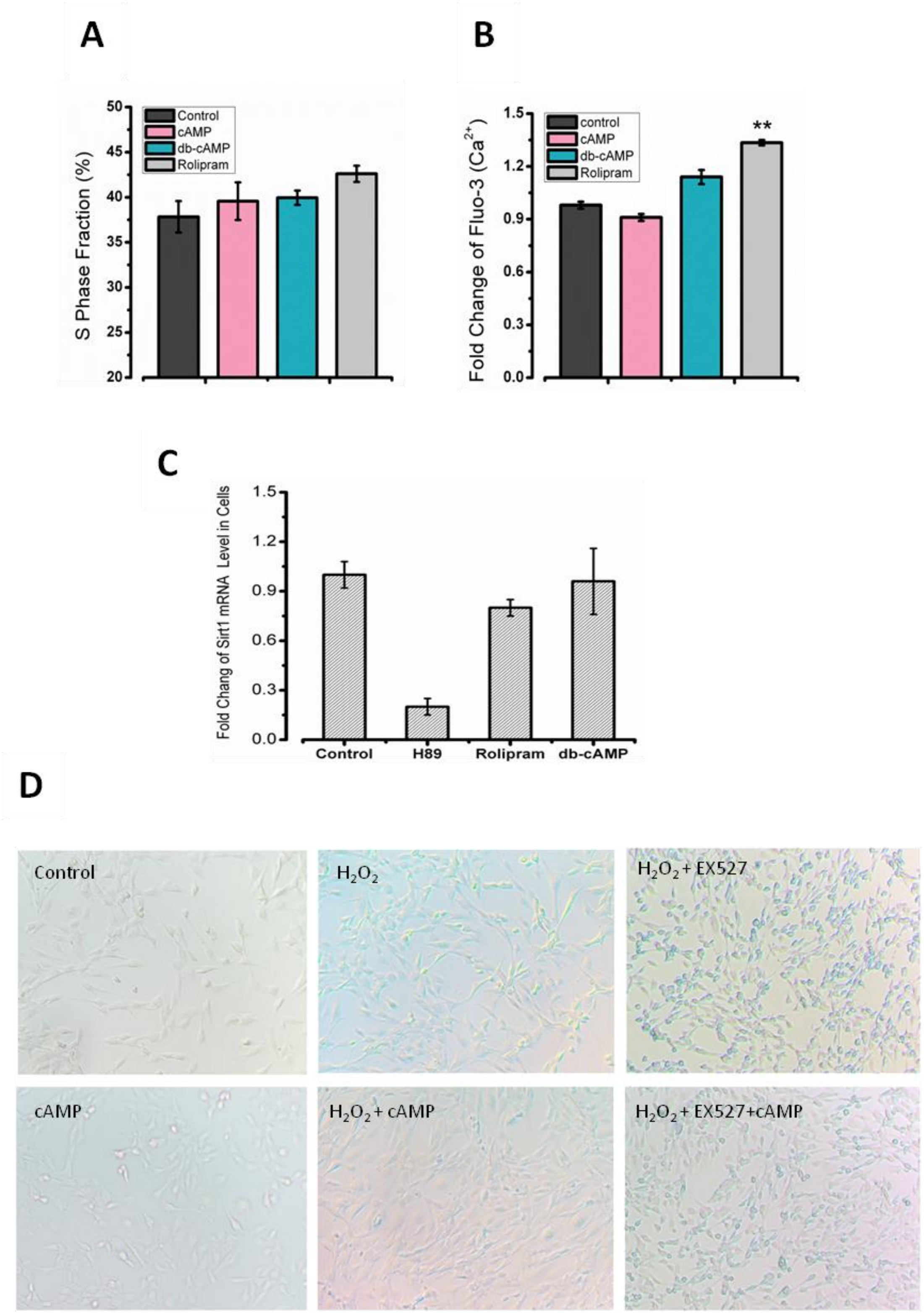

Figure S4 cAMP Promotes Sirtuin to Prevent Oxidative Damage and Cell Senescence

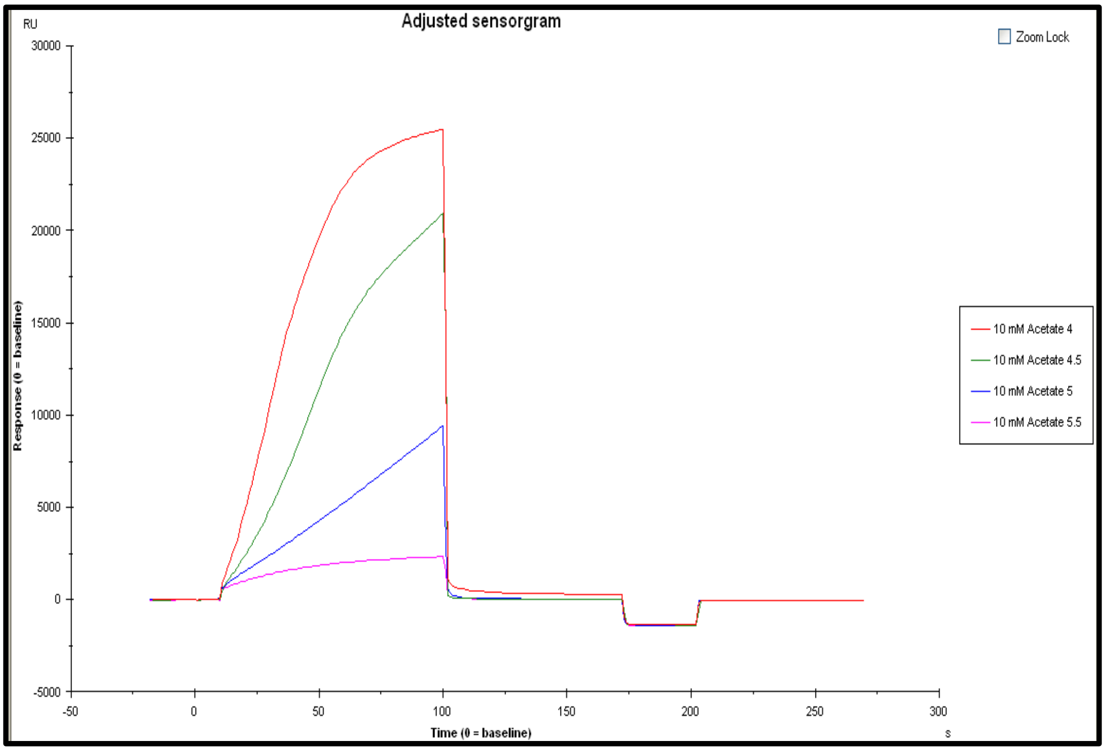

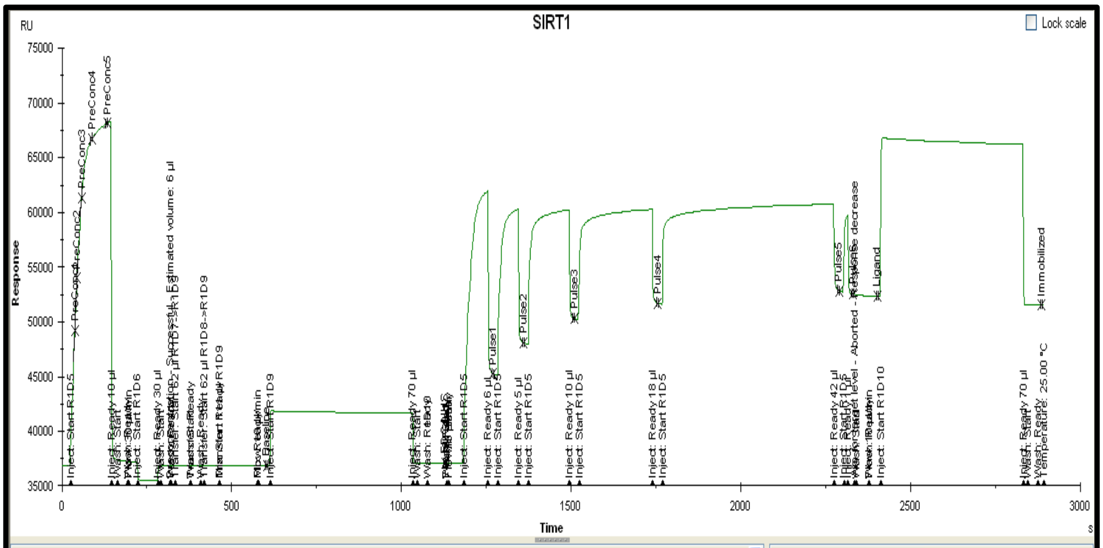

Fc	Time	Window	AbsResp	SD	LRSD	Slope	RelResp	Baseline	Id
2	133.0	5	68131.7	35.73	0.46	19.10	#NA	No	PreConc5
2	603.0	5	36914.3	0.02	0.00	0.01	0.0	Yes	Baseline
2	1128.0	5	37108.7	0.92	0.91	-0.22	194.4	No	EDC/NHS
2	1270.0	5	45274.5	34.11	3.71	-18.15	8360.2	No	Pulse1
2	1360.0	5	48089.0	27.55	2.68	-14.67	11174.7	No	Pulse2
2	1509.0	5	50332.7	31.92	3.10	-17.00	13418.4	No	Pulse3
2	1755.0	5	51728.1	31.54	2.82	-16.80	14813.8	No	Pulse4
2	2289.0	5	52867.3	40.20	3.48	-21.42	15953.0	No	Pulse5
2	2331.0	5	52544.1	13.82	0.94	-7.37	15629.8	No	Pulse6
2	2402.0	5	52333.1	3.07	0.02	-1.64	15418.8	No	Ligand
2	2886.0	5	51578.2	0.64	0.01	-0.34	14663.9	No	Immobilized

Keywords in cycle 2	Value
Chip	CM5
Ligand	SIRT1
Method	Amine
Procedure	TargetLevel
TargetLevel	20000

Online - COM1 | Temperature: 25.00 °C | Sensor chip: CM5

Sample compartment temperature - current: 25 °C set: 25 °C | Running standby, remaining time: 7.0 days

Figure S5. The Immobilization Result of SIRT1 on CM5 Chip for SPR analysis.

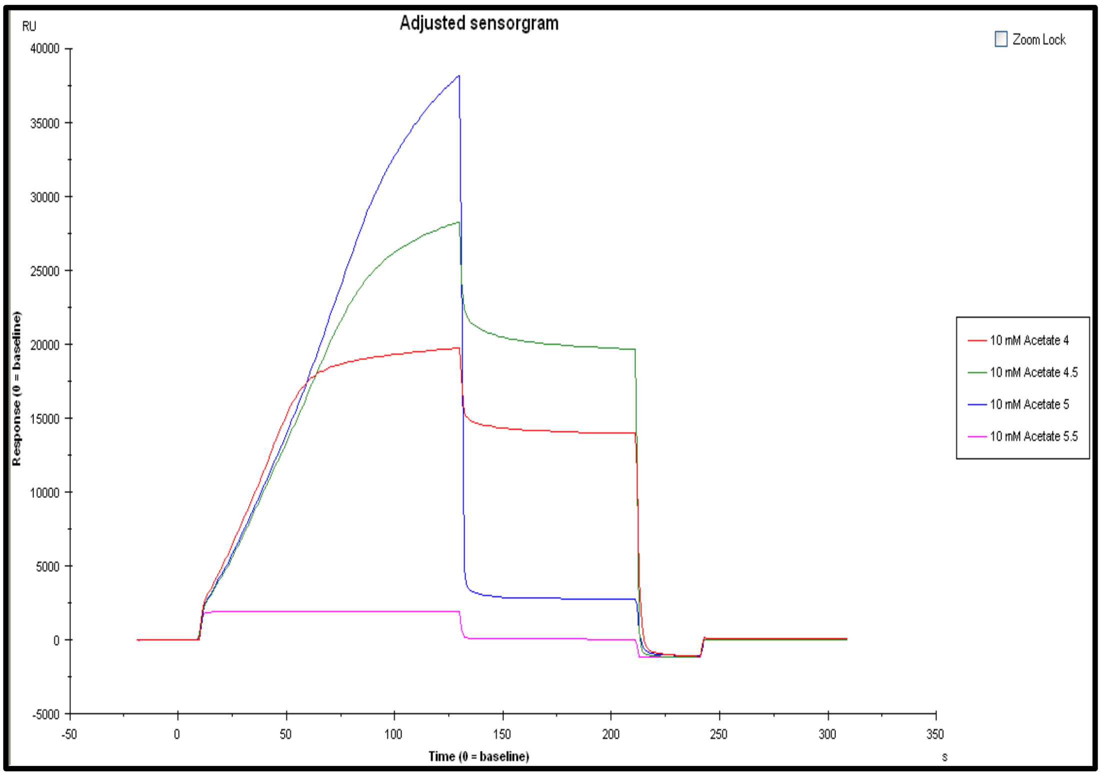

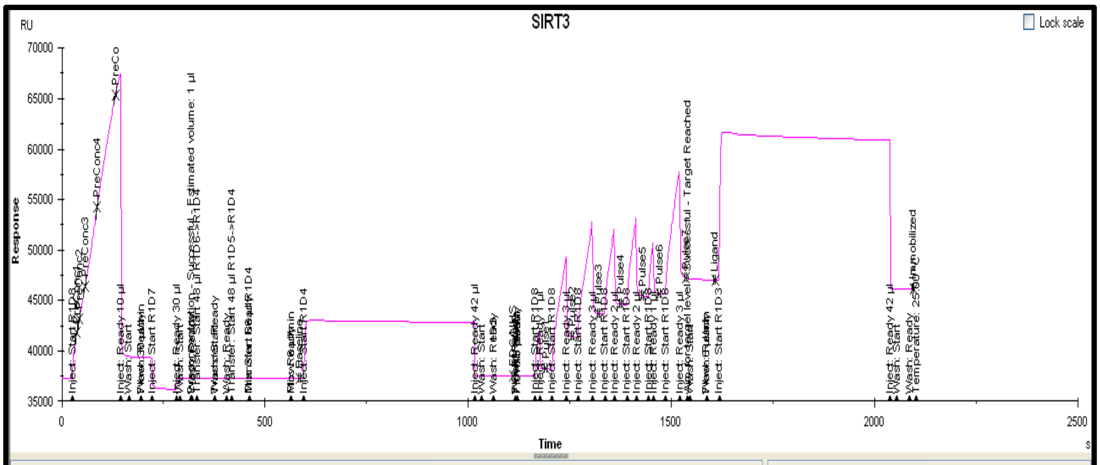

Fc	Time	Window	AbsResp	SD	LRSD	Slope	RelResp	Baseline	Id
4	37.0	5	41689.8	421.98	3.67	225.55	#NA	No	PreConc1
4	43.0	5	43028.9	416.70	1.66	222.74	#NA	No	PreConc2
4	57.0	5	46275.0	454.12	3.36	242.73	#NA	No	PreConc3
4	87.0	5	54101.5	510.28	0.38	272.76	#NA	No	PreConc4
4	132.0	5	65238.9	361.82	4.54	193.39	#NA	No	PreConc5
4	585.0	5	37332.9	0.01	0.01	0.00	0.0	Yes	Baseline
4	1110.0	5	37502.0	1.14	0.77	-0.48	169.1	No	EDC/NHS
4	1191.0	5	38209.8	3.53	0.26	-1.88	876.9	No	Pulse1
4	1256.0	5	41420.4	12.39	1.25	-6.59	4087.5	No	Pulse2
4	1321.0	5	43715.6	19.09	1.66	-10.18	6382.8	No	Pulse3
4	1375.0	5	44634.6	16.60	1.29	-8.85	7301.7	No	Pulse4
4	1429.0	5	45493.9	18.29	1.40	-9.75	8161.0	No	Pulse5
4	1470.0	5	45676.9	12.63	0.91	-6.74	8344.0	No	Pulse6
4	1536.0	5	47265.7	26.26	2.10	-14.00	9932.8	No	Pulse7
4	1607.0	5	47014.9	2.33	0.04	-1.24	9682.0	No	Ligand
4	2095.0	5	46123.6	0.67	0.03	-0.36	8790.7	No	Immobilized

Keywords in cycle 2	Value
Chip	CM5
Ligand	SIRT3
Method	Copy of Amine
Procedure	TargetLevel
TargetLevel	9000

Online - COM1 | Temperature: 25.00 °C | Sensor chip: CM5

Sample compartment temperature - current: 25 °C set: 25 °C | Running standby, remaining time: 7.0 days

Figure S6. The Immobilization Result of SIRT3 on CM5 Chip for SPR analysis.

Table S1. Binding Energy for Different Docking conformation.

Mode	affinity(kcal/mol)	dist from best mode	(rmsd l.b.| rmsd u.b)
1	-10.2	0.000	0.000
2	-10.0	1.093	2.150
3	-9.1	2.012	3.564
4	-8.9	2.177	2.700
5	-8.9	6.432	9.085
6	-8.8	7.836	10.054
7	-8.7	1.043	1.966
8	-8.6	9.506	11.875
9	-8.5	5.634	8.304
10	-8.2	5.368	6.852
11	-8.1	5.208	6.749
12	-8.1	5.195	6.875
13	-8.0	4.879	7.154
14	-7.8	5.030	6.712
15	-7.7	4.734	6.845
16	-7.5	8.664	11.161
17	-7.5	4.284	6.169
18	-7.5	4.207	7.146
19	-7.3	4.097	6.066
